# Inflammatory Bowel Disease in Children and Young People Living With Overweight or Obesity: A Critical Narrative Review

**DOI:** 10.1111/obr.70063

**Published:** 2025-12-09

**Authors:** Razan Algarni, Efstathia Papada, Adrian Brown

**Affiliations:** ^1^ Division of Medicine University College London London UK; ^2^ Department of Clinical Nutrition, College of Applied Medical Sciences Imam Abdulrahman Bin Faisal University Dammam Saudi Arabia; ^3^ Centre for Obesity Research University College London (UCL) London UK; ^4^ Bariatric Centre for Weight Management and Metabolic Surgery University College London Hospital NHS Trust (UCLH) London UK; ^5^ UCLH NIHR Biomedical Research Unit London UK

**Keywords:** children, Crohn's disease, inflammatory bowel disease, obesity, overweight, ulcerative colitis, young people

## Abstract

Inflammatory bowel disease is a chronic inflammatory disorder affecting the gastrointestinal tract. It mainly comprises of Crohn's disease and ulcerative colitis. Its global prevalence has risen simultaneously with overweight and obesity among children and young people over the last decades. This critical narrative review aims to explore how living with overweight or obesity contributes to the development of inflammatory bowel disease (IBD) as well as the disease course in children and young people. Approximately, 24% of children and young people with the disease are living with overweight or obesity, with the majority being diagnosed with ulcerative colitis. Obesity appears to be associated with increased disease activity, adverse effects, with increased adiposity contributing to IBD pathogenesis though multiple mechanisms. Here, we offer a novel mechanistic pathway to how obesity impacts on IBD pathogenesis. Increased adiposity appears to contribute to its pathogenesis through increased proinflammatory cytokines and microbiota imbalance leading to inflammation and increased intestinal permeability. Additionally, adiposity appears to exacerbate micronutrient deficiencies associated with inflammatory bowel disease, including iron and vitamin D. Obesity is also shown to be associated with increased disease activity. Finally, we review possible weight management interventions available to children and young people living with obesity and overweight and IBD. In conclusion, living with overweight and obesity are proposed to have adverse effects on inflammatory bowel disease in children and young people, with an urgent need for greater research to better understand their impacts and assist in guiding effective tailored interventions.

## 1 Introduction

Inflammatory bowel disease (IBD) is a chronic inflammatory disorder of the gastrointestinal tract including two main types, Crohn's disease (CD) and ulcerative colitis (UC) [1]. CD is characterized by inflammation involving all layers of the bowel wall, which can affect any part of the gastrointestinal tract. In contrast, UC is characterized by damage and inflammation limited to the mucosal layer and affecting the colon only [1].

Global incidence and prevalence of IBD in children and young people is highest in Northern Europe and North America [2]. IBD incidence increased by 94% among adolescents between 2000 and 2017 [3] with a twofold increase in IBD incidence among children and young people seen in the last 20 years, including in England [4].

The exact causes of IBD in children and young people are still not fully understood but seem to include gene mutations, which lead to altered gastrointestinal tract immune homeostasis [5]. However, the variation in gene expression among individuals carrying associated genes indicates the crucial role of gene–environment interactions in the development of IBD [6].

In parallel to the increase in IBD cases seen among children and young people in the last years [7], obesity prevalence also more than doubled in many countries between 1990 and 2022 in school‐aged children and young people [8]. Interestingly, although the highest rates of IBD are reported in Northern Europe and North America, the highest prevalence of overweight and obesity was reported in North Africa and the Middle East, and the greatest increase from 1990 to 2021 was seen in Southeast Asia, East Asia, and Oceania. By 2021, females in many countries in Australasia and in high‐income North America were living with obesity predominantly [9]. Recent studies show a significant increase in the prevalence of overweight and obesity among adults, children, and young people living with IBD, which is contrary to the traditional belief [10]. Globally, approximately 15%–40% of patients with IBD live with obesity, and 20%–40% live with overweight [11]. This led to the hypothesis that living with overweight and obesity might contribute to the development and pathogenesis of IBD [11].

There is currently limited or conflicting evidence on the impact of overweight and obesity on IBD pathogenesis, prognosis, and response to treatment [12], particularly in children and young people. Therefore, the present review aims to explore the current available data on overweight and obesity in children and young people diagnosed with IBD to examine whether living with overweight or obesity is associated with increased risk of IBD development and disease pathogenesis. Moreover, this review explores whether living with overweight or obesity negatively impacts the clinical course and prognosis of the disease.

## 2 Methodology

MEDLINE and PubMed databases were searched for related studies from January 2005 to December 2024. Search terms included “inflammatory bowel disease,” “overweight,” “obesity,” “children,” “young people,” “adolescents.” Studies were initially excluded based on titles and abstracts. Relevant studies were reviewed and synthesized. Studies were also extracted from the references of other included studies. As this is a narrative review, a systematic literature search was not conducted; rather, this paper aimed to map relevant literature. However, due to limited available mechanistic studies on children and young people, studies on adults were also considered to help understand the pathogenesis.

## 3 Obesity Prevalence and IBD in Children and Young People

Multiple studies have shown a significant prevalence of overweight and obesity among children and young people living with IBD (Table 1), with up to 24% of children newly diagnosed with IBD living with overweight or obesity [14]. Central obesity may play a role, as data show that Finnish school‐aged children living with central obesity are at a two times higher risk of developing several immune‐mediated diseases, including IBD [21]. Most studies show that living with overweight and obesity is more likely to be prevalent among children and young people diagnosed with UC or indeterminate colitis (IC) [14] as compared to CD. This finding is contrary to what is seen in the adult population diagnosed with IBD, where living with overweight or obesity is mostly linked to CD rather than UC [22], thus raising the question of whether obesity and increased adiposity are associated with different IBD types among different age groups.

**TABLE 1:** Prevalence of overweight and obesity in children and young people living with inflammatory bowel disease.

Study type	Age	Country/region	Prevalence of overweight and obesity in children and young people living with IBD			Comments
			IBD	CD	UC	
Retrospective study Kugathasan et al. 2007 [13]	4–16 years	North America		10%	20%–30%	Percentage included those at risk of living with overweight as well
Retrospective study Long et al. 2011 [14]	2–18 years	United States	24%	20%	30%	IC was included among the UC percentage
Retrospective study Pituch‐Zdanwoska et al. 2015 [15]	0–18 years	Poland	8.4%	4.3%	13.4%	Percentage included those living with overweight or obesity
Retrospective study El Mouzan et al. 2020 [16]	0– < 18 years	Saudi Arabia	13%	9%	20%	Percentage included those living with overweight or obesity
Retrospective study Carbonell and Chandan 2020 [17]	2–17 years	United States	11.6%	8%	15.7%	Percentage included those living with overweight or obesity
Prospective observational study Chandrakumar et al. 2020 [18]	< 17 years	Canada	17%	10%	21.5%	All patients with CD were living with overweight
Retrospective study Yerushalmy‐Feler et al. 2021 [19]	12–16 years	Tel‐Aviv, Isreal	11.2%	4.7%	19.4%	Percentage included those living with obesity only
Retrospective study Jain et al. 2022 [12]	2–19 years	United States	19%	16%	27%	Percentage included those living with overweight or obesity
Prospective study von Graffenried et al. 2022 [20]	0–18 years	Switzerland	12.8%	12.4%	14%	Percentage included those living with overweight or obesity

Abbreviations: % = percentage, CD = Crohn's disease, IBD = inflammatory bowel disease, IC = indeterminate colitis, UC = ulcerative colitis.

Furthermore, children and young people with IBD and living with overweight experience significantly longer durations of symptoms prior to diagnosis [23]. A possible explanation for this may be the exclusion of an IBD diagnosis in individuals with overweight or obesity due to the traditional belief that IBD is associated with underweight individuals [10]. Therefore, this may lead to a reduction in body weight due to malabsorption and reduced food intake by the time individuals are diagnosed [23]. This may also lead to an underestimation of the prevalence of overweight and obesity among this population.

## 4 Obesity and IBD Pathogenesis in Children and Young People

Obesity, which is characterized by a state of continuous low‐grade chronic inflammation [24], is thought to have a role in the pathogenesis of IBD through several mechanisms involving dysbiosis, adipose tissue, and altered intestinal permeability [10]. Moreover, both obesity and IBD are characterized by gut dysbiosis, bacterial translocation, and inflammation [25] and therefore potentially share some similar mechanisms of pathogenesis. However, most of the evidence to date is from studies on adults or preclinical studies [14].

### 4.1 Westernized Lifestyle, Obesity, and Altered Microbiota

Obesogenic environments are characterized by decreased physical activity [26] and Westernized diets. These types of diets are typically energy dense and often rich in processed foods that have a high content of refined carbohydrates, salt, and saturated fats, are low in fiber, and have been associated with the rise in the obesity epidemic [27]. Apart from the direct effects on obesity, they seem to exert indirect effects through the gut microbiota [26]. High‐fat diets directly, and obesity associated with a high‐fat diet, have been linked to altered gut microbiota [28]. Mice that were fed a high‐fat diet were shown to have an altered gut microbiota even prior to the development of obesity. They had high *Firmicutes* and low *Bacteroidetes, as Firmicutes* are characterized by increased energy absorption from food, which might contribute to the development of obesity. This increase in the level of *Firmicutes* following the administration of the obesogenic diet was slower in females as compared to males [29]. Additionally, additives including emulsifiers and artificial sweeteners were shown to have a possible association with dysbiosis and obesity [30]. Increased intake of emulsifiers was shown to cause an altered microbiome balance and contribute to increased weight and adiposity, leading to increased inflammatory markers in animal models [30]. In vitro studies suggest that this could be through altered microbiota gene expression with increased flagella expression and lipopolysaccharide (LPS) levels, which are linked to higher energy storage, ultimately driving intestinal inflammation [31]. Nevertheless, although exclusive enteral nutrition (EEN) containing emulsifiers has proven effective in inducing remission in Crohn's disease [32], additional research is warranted to better understand the impact of emulsifiers on intestinal inflammation. Similarly, some artificial sweeteners were associated with altered gut microbiota such as acesulfame potassium, which has been shown to disrupt the gut bacterial composition and induce weight gain and associated chronic inflammation in mice, possibly through alterations in bacterial energy metabolism [33]. Nevertheless, the evidence from pediatric studies is limited and suggests a positive association between increased BMI in children that consume artificially sweetened carbonated soft drinks, weight gain, increased body fat accumulation, and obesity. The observational nature of the majority of studies could not confirm a direct causality of artificial sweeteners consumption with weight gain [34]. Sucralose is also associated with altered gut microbiota and increased tissue inflammation through altered bacterial genes associated with increased proinflammatory markers [35]. Moreover, low dietary fiber intake is associated with decreased microbiota diversity, which might not be reversible in later generations [36]. Taken together, the impact of the changing food environment appears to potentially place people at greater risk of developing IBD.

Apart from the effects of diet on the risk for IBD, there is emerging evidence on the importance of dietary treatment for patients with IBD with several dietary regimes and supplements being investigated. For example, the Crohn's Disease Exclusion Diet (CDED), combined with partial enteral nutrition (PEN) providing 50% of energy needs, consists of three phases, with the first including a 6‐week induction therapy for children and adults with Crohn's disease. It includes a polymeric oral supplement, five mandatory daily foods, 14 permitted foods, and excludes all others [32]. In Phases 2 and 3, the diet is gradually liberalized. The rationale of CDED was based on the elimination of foods and additives that may harm the gut microbiota or compromise intestinal barrier function [37], given their role in IBD pathogenesis, as discussed above.

### 4.2 Dysbiosis, Obesity, and IBD

Dysbiosis is characterized by an imbalance in the number or diversity of microbes in the gut, leading to abnormal composition and function of the microbiota, which negatively affects the host homeostasis [38]. Twin studies have shown that gut microbiota is largely influenced by genetic factors but also by other environmental factors including diet, medication, and anthropometric measurements [39].

Dysbiosis seems to have a bidirectional relationship with obesity through several mechanisms including immune dysregulation, proinflammatory effects, and altered regulation of energy and gut hormones [40]. This may lead to overweight or obesity through increased appetite and storage of adipose tissue, increased absorption of energy, and chronic inflammation [41]. Children and young people living with obesity show differences in gut microbiota composition compared to those with normal weight, such as a greater proportion of *Firmicutes* compared to *Bacteroidetes* [42]. This could explain higher carbohydrates' fermentation in young adults living with obesity compared to lean individuals [43]. However, a meta‐analysis showed that the gut composition of *Firmicutes* and *Bacteroidetes* is inconsistent among individuals with obesity potentially due to genetics and environmental factors, unlike IBD, where consistency has been shown in gut bacterial composition [44]. Moreover, a previous study showed that gut microbiota in children and adolescents aged 7–18 years was significantly affected by body mass index (BMI) and other lifestyle factors including diet and exercise, where gram‐negative phylum *Proteobacteria* was associated with obesity [45]. Gram‐negative bacteria are known to produce proinflammatory markers including LPS that increase fat and energy storage, suggesting their potential role in overweight and obesity [43]. On the other hand, beneficial bacteria species such as *Bifidobacterium* were shown to reduce obesity‐associated systemic and adipose tissue inflammation in mice with diet‐induced obesity [46].

Similarly, dysbiosis is also shown to be present in adults with IBD including increased *Proteobacteria*, which have proinflammatory effects on the host [47]. This is likewise observed in children and young people living with IBD, where those with increased levels of *Proteobacteria* were significantly more likely to have severe IBD outcomes, such as structuring or penetrating disease as compared to those with lower levels [48]. Dysbiosis has been linked to abnormal mucosal immune responses, intestinal barrier dysfunction, and increased porosity. These result in increased intestinal permeability [49], which is linked to the translocation of bacterial products to systemic circulation, including bacterial LPS, also shown to be elevated in individuals living with obesity [50]. Bacterial translocation has been linked with chronic inflammation of adipose tissue, insulin resistance, and metabolic syndrome, all of which have been associated with overweight and obesity as well [49], suggesting an association between dysbiosis, obesity, and IBD development. On the other hand, beneficial bacteria such as *Bifidobacterium* are shown to maintain normal gut permeability and reduce proinflammatory cytokines in mice with colitis [51].

## 5 Adipose Tissue, Inflammation, and Implications in IBD

Adipose tissue has long been known for its significant role in energy storage, particularly white adipose tissue (WAT), which is found throughout the human body but mainly viscerally and subcutaneously. It represents the primary source of free fatty acids (FFA) in postprandial states for energy and heat production [52]. In addition to the vital role of adipose tissue in energy storage, it is also considered the body's largest endocrine organ [52]. It is responsible for secreting hormones, cytokines, and proteins, which affect cells and tissue functions, regulate metabolic functions, and have pro‐ and anti‐inflammatory effects [53]. The secretion of proinflammatory mediators including interleukins (IL), tumor necrosis factor alpha (TNF‐α), interferon gamma (IFN‐γ), and acute phase proteins such as C‐reactive protein (CRP) [54] alongside immune cell infiltration, is shown to be increased in the adipose tissue of individuals living with obesity compared to that of lean individuals [53]. Moreover, obesity is associated with increased CRP levels in adult patients living with IBD, as reported by a retrospective study of prospectively collected data over a period of 3 years, where rates of CRP increase differed significantly between the different BMI subgroups. The highest rates of CRP elevation were observed in patients with obesity type III (BMI ≥ 40 kg/m^2^) [55].

The imbalance in the secretion of adipocytokines in individuals living with overweight or obesity leads to an overall increase in the levels of inflammatory cytokines, which can trigger innate immune responses and low‐grade inflammation [53]. This proinflammatory state has been linked to disrupted intestinal mucosa and increased intestinal permeability. Increased intestinal permeability, which is a shared characteristic of both dysbiosis and the proinflammatory state seen with increased adipose tissue, leads to a further increase in fat‐derived inflammatory adipocytokines, T‐cell infiltration, and bacterial translocation. This seems to be involved in the pathogenesis of IBD [54] as shown in animal studies where intestinal permeability was associated with the development of IBD [56].

## 6 Sarcopenic Obesity and IBD Activity

Altered body composition (bc) in patients with IBD is thought to be associated with altered metabolism caused by chronic inflammation, increased energy expenditure, reduced energy intake, malabsorption, and reduced physical activity [57]. Sarcopenia is defined as a general skeletal muscle disorder characterized by a decrease in the mass and function of skeletal muscle, which affects the strength and performance of muscle [57]. Although it was originally associated with aging, it may also be related to inflammation‐mediated chronic diseases and relevant medical treatments, including the pediatric population [57]. When developed at younger ages, it may impact growth, nutritional, and psychological status [58].

Sarcopenic obesity (SO) is characterized by the presence of sarcopenia and increased fat mass concurrently [59]. It is shown to be associated with inflammation and other non‐communicable diseases [59]. Moreover, SO, defined as sarcopenia together with a body fat percentage > 27% and > 38% in men and women, respectively, was found to be prevalent in adults living with IBD [59] and was associated with adverse IBD outcomes, including frequent disease relapse and rehospitalization compared to those without sarcopenic obesity [59]. In addition, sarcopenia, defined with a skeletal muscle mass of < 38.5 cm^2^/m^2^ for women and < 52.4 cm^2^/m^2^ for men, was significantly associated with the increased need for IBD associated surgery in adults living with increased weight (BMI > 25 kg/m^2^) [60]. This is thought to be associated with poor nutritional intake, altered absorption, and intestinal mucosal impairment, leading to increased inflammatory responses and poor nutritional status, contributing to the catabolic state seen in IBD [61]. Moreover, a meta‐analysis showed that adults with IBD and sarcopenia or SO had higher CRP levels as compared to those without sarcopenia [62].

In a systematic review assessing bc in children and young people living with IBD, most patients with CD and UC had decreased lean mass (LM) and fat‐free mass (FFM) independent of their weight when compared to healthy individuals, which were shown to be associated with increased disease activity [63]. These abnormalities were noticed even in remission states and in cases where BMI was increased, which supports findings of other studies on adult patients relating IBD with reduced LM even in the presence of weight gain [63]. Mechanistically, chronic inflammation has been suggested to be associated with sarcopenia leading to IBD [64]. Furthermore, reduced quality of life and fatigue associated with muscle mass loss may also contribute to IBD progression [65, 66]. However, the mechanisms implicated in sarcopenia leading to IBD should be further explored.

## 7 Vitamin D Deficiency, Obesity, and IBD

Vitamin D deficiency prevalence is high among children and young people living with overweight and obesity as compared to those with a healthy weight [67]. Some evidence suggests its bidirectional relationship with obesity. Vitamin D deficiency might be a risk factor for the later development of obesity in school‐aged children [68], possibly due to the adipogenesis‐inhibiting role of vitamin D through its effect on decreasing peroxisome proliferator‐activated receptor‐gamma (PPAR‐gamma) availability [69]. Furthermore, vitamin D deficiency is present in a significant proportion of children and young people living with IBD, with a higher prevalence among those with UC [70], which might be due to poor oral intake, poor absorption, and altered metabolism [70]. Vitamin D deficiency may play a role in chronic inflammation associated with increased adipose tissue through its potential role in inducing adiposity, thereby contributing to the dysregulation of the intestinal barrier and IBD development.

## 8 Potential Mechanisms Linking Obesity and IBD

**FIGURE 1:**
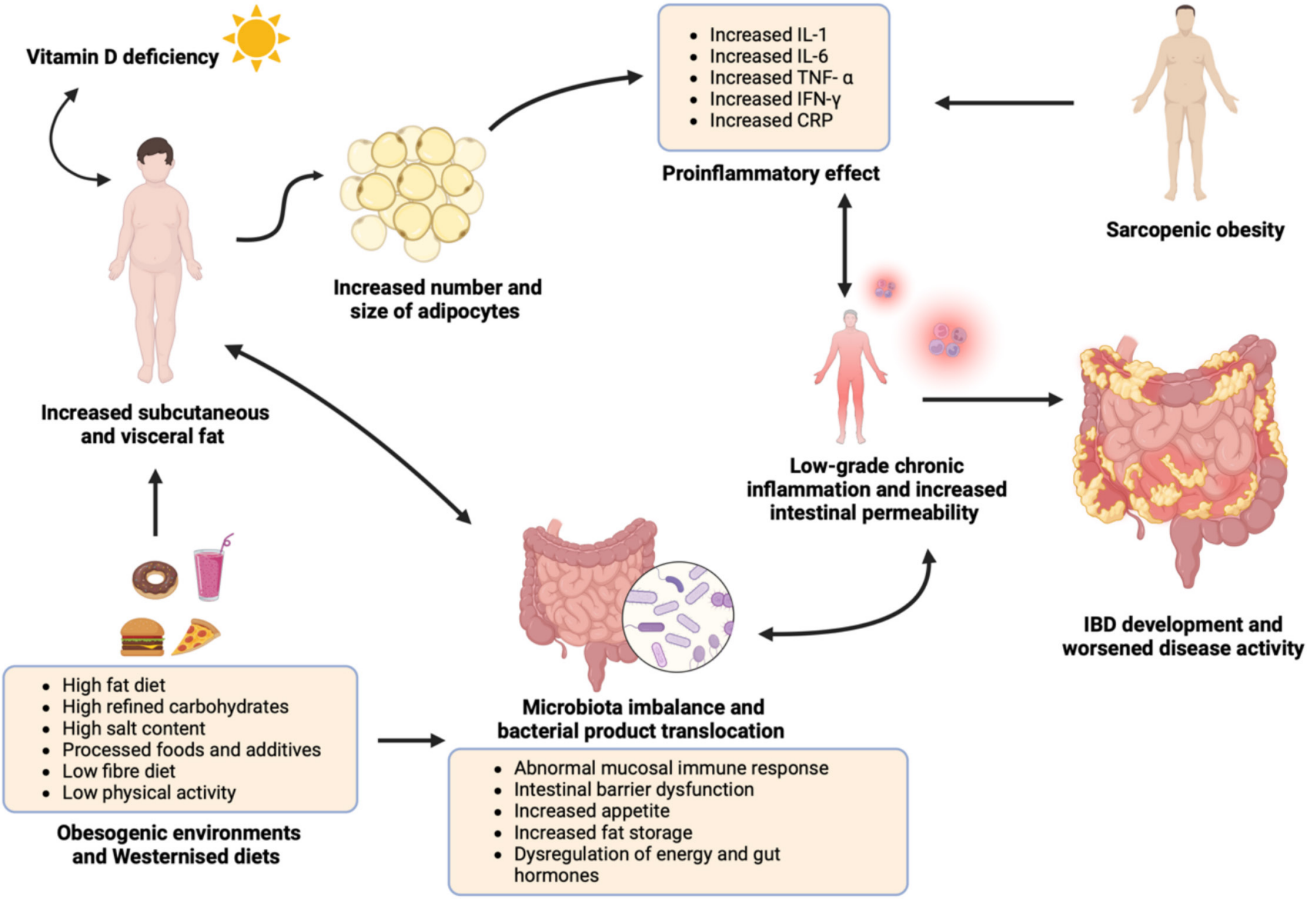
Obesogenic environments, sedentary lifestyles, and Westernized diets characterized by high contents of fat, refined carbohydrates, processed foods, and additives and low in fibers and lower physical activity are shown to be associated with the development of overweight and obesity directly and through their negative effect on gut microbiota leading to increased body fat mass. Vitamin D deficiency appears to have a bidirectional relationship with obesity and IBD and play a role in increased adiposity. In children and young people weight gain is characterized by an increase in both the number and size of adipocytes. The increased adipose tissue exposes the body to a proinflammatory state by increasing the secretion of proinflammatory mediators including IL‐1, IL‐6, TNF‐α, IFN‐γ, and CRP. Together, increased adipose tissue and dysbiosis might contribute to low grade‐chronic inflammation and immune induced barrier dysfunction, leading to increased intestinal permeability and ultimately IBD. Sarcopenic obesity is shown to worsen IBD activity through its proinflammatory effect. These complex interactions suggest the potential obesity‐associated mechanisms leading to IBD pathogenesis. Figure created with BioRender.com. CRP = C‐reactive protein, IBD = inflammatory bowel disease, IFN‐γ = interferon gamma, IL‐1 = interleukin‐1, IL‐6 = interleukin‐6, TNF‐α = tumor necrosis factor alpha.

IBD is thought to develop in genetically susceptible individuals due to complex interactions between the immune system, gut microbiome, and environment, all of which have been associated with obesity as well [71]. This may explain the association of Westernized lifestyles and increased prevalence of overweight and obesity with IBD development and pathogenesis. This is possibly through the interaction of altered gut microbiota and activation of immune responses, which may further be exacerbated in individuals living with overweight or obesity, leading to chronic inflammation. Both dysbiosis and chronic inflammation of adipose tissue, which are common characteristics of obesity, are shown to be associated with bacterial translocation and intestinal barrier dysfunction, leading to increased intestinal permeability and possibly IBD development. However, despite the current evidence linking obesity with IBD, more human studies are needed to understand the mechanisms linking overweight and obesity with IBD in children and young people [54]. Proposed mechanisms that associate obesity with IBD pathogenesis are presented in Figure 1.

## 9 Effects of Overweight and Obesity on the Clinical Course and Prognosis of IBD in Children and Young People

Studies investigating the clinical course and prognosis of IBD among children and young people living with overweight and obesity are limited [12]. Both lower and upper BMI quartiles were associated with increased disease activity at the time of IBD diagnosis in children and young people living in Tel Aviv, Israel, which was identified by the Pediatric Crohn's Disease Activity Index (PCDAI) or the Pediatric Ulcerative Colitis Activity Index (PUCAI) [23]. Moreover, they were associated with disease exacerbation, which was identified as relapse after remission and elevated scores in PCDAI or PUCAI. This was also observed in another study in a Hungarian population, which showed that disease activity was associated with obesity in newly diagnosed pediatric patients with UC [72].

This increased IBD disease activity among children and young people living with overweight or obesity might be associated with the delayed diagnoses due to the exclusion of IBD diagnosis in this population among healthcare providers, as discussed earlier [23]. Another possible cause is the inflammatory effect of obesity through increased adipocytes, as they are responsible for releasing proinflammatory mediators including TNF‐α, which has been linked to IBD and might contribute to increased disease activity [54]. This could also be associated with the increased use of anti‐TNF therapy for the management of IBD among children and young people living with excess weight [23].

On the other hand, there were no significant differences in time to first relapse, IBD‐associated hospitalization, and medical or surgical management between children and young people living with overweight or obesity compared to those with normal or underweight [18, 23]. Furthermore, no differences were seen in disease activity, which was assessed by physician global assessment (PGA), PCDAI for CD, or PUCAI for UC between the two groups [18]. Similarly, no significant differences were seen in disease severity, rates of surgical interventions, short‐term clinical outcomes, or length of hospital stay between children and young people with overweight or obesity compared to those with normal or underweight. Indeed, those with overweight or obesity did not seem to have a higher risk of developing complications such as anemia due to blood loss, percutaneous drainage, or other postoperative complications than their counterparts. However, they were at a higher risk of developing central venous catheter infections [73]. Another multicentered cohort population of children and young people from the United States showed similar disease activity at 1‐year follow‐up between those with overweight or obesity and those with normal weight [12].

The effect of overweight and obesity on outcomes of IBD among Swiss children and adolescents was similarly investigated in a large cohort study. The findings supported other studies that showed there were no significant differences in remission rates and frequency of hospitalization between those living with overweight and obesity compared to those with healthy or underweight within the past 12 months. Extraintestinal manifestations were also similar among the two groups except for arthritis, which was significantly higher among children and adolescents living with obesity as compared to those with normal weight in the CD group. PUCAI scores showed that clinical disease activity was higher among those living with obesity. However, no differences were seen between the two groups in terms of biological activity, which was measured by CRP levels, calprotectin, frequency of bloody stools, or extraintestinal manifestations [20].

## 10 Effects of Overweight and Obesity on the Response to IBD Treatment in Children and Young People

Research investigating the effect of excess weight on the medical management and response to treatment in children and young people living with IBD is likewise limited. Overweight and obesity did not seem to affect the response to anti‐TNF therapy or influence the dependence on corticosteroid therapy in children and young people living with overweight or obesity as compared to those with normal or underweight in Canada [18]. Furthermore, there were no significant differences in the use of corticosteroids, 5‐aminosalicylate, methotrexate, thiopurine, or anti‐TNF medications between children and young people living with overweight or obesity compared to those with normal weight at 1‐year follow‐up post IBD diagnosis in a population from the United States [12]. Although no associations were seen between the BMI of children and adolescents with IBD living in Tel Aviv and treatments with immunomodulators or corticosteroids, the use of anti‐TNF therapy was however increased among those with a BMI in the lower or upper quartiles [23]. On the other hand, a study evaluating the effect of overweight and obesity at the start of anti‐TNF therapy on treatment response and relapse rate in children with IBD showed that children living with overweight and obesity required more frequent dose escalations. Overall loss of response to anti‐TNF therapy was not increased, but in the long term, they tend to have a higher risk for relapse [74]. This is further supported in another recent study that showed that high BMI in children and young people diagnosed with CD was associated with anti‐TNF treatment failure, specifically adalimumab. Although no differences were observed in clinical outcomes or drug levels with infliximab, probably due to weight‐based dosing and proactive drug monitoring [75].

Studies assessing the effects of overweight and obesity on IBD‐associated surgical outcomes in children and young people remain limited. Children and young people living with overweight and obesity and diagnosed with CD had significantly higher rates of surgeries due to perianal abscesses, although there was no association seen among those with UC [20]. However, other studies on similar populations showed that there were no associations between overweight and obesity and increased need for IBD‐associated surgery as compared to those with normal weight in North America [18, 73]. Despite the conflicting results, managing overweight and obesity, under healthcare professional supervision, should be considered in the preoperative and postoperative care of children and young people due to the potential complications associated with excess weight in surgical procedures [76]. These include recommended equipment for children living with overweight and obesity, for example, extra‐large pediatric gowns, suitable chairs and trolleys, and postoperative respiratory complications.

## 11 Effects of Weight Loss Interventions on Outcomes of IBD

Overweight and obesity interventions are important for all children and young people living with excess weight, including those diagnosed with IBD [14]. Some IBD medical treatments, including anti‐TNF‐α and corticosteroids, have been linked to weight gain in children and adolescents, which raises the importance of monitoring body weight and providing appropriate counseling and education regarding lifestyle modification for the prevention and management of overweight and obesity [77] prior to their commencement. However, despite the suggested proinflammatory effects of overweight and obesity on the clinical course of IBD and its possible impact on response to therapy, studies evaluating the effect of weight loss interventions on outcomes of IBD are sparse [78]. It is important to highlight that weight loss should only be advised during periods of stable remission, due to the increased risk of micronutrient deficiencies, sarcopenia, and SO in this population, especially during active disease periods characterized by a catabolic state [79].

Weight management in children living with overweight or obesity who have not reached their adult height yet aims for them to grow into their weight rather than to lose it. When adult height is achieved in adolescence, excess weight should be lost [80]. Weight management interventions for children and young people primarily include dietary and lifestyle modifications [80], though pharmacotherapy and bariatric surgery are considered following specialist multidisciplinary team (MDT) discussions. The National Institute for Health and Care Excellence (NICE) suggests referring children and young people living with overweight and obesity (≥ 91st centile) and those with comorbidities to appropriate specialist services that might offer more intensive interventions. These include behavioral and psychological care, pharmacotherapy for adolescence, and bariatric surgery for young people with special circumstances through a multidisciplinary approach. Weight loss should be achieved under the guidance of a specialist dietitian due to increased risk of muscle loss and micronutrient deficiency if weight loss is attempted under no guidance [79] and a wider MDT. Additionally, some patients with IBD might not tolerate specific types of fruits and vegetables that are considered a fundamental part of a healthy diet, especially during periods of relapse, which raises the importance of receiving specialist dietetic care [78].

Studies assessing the effects of pharmacotherapy associated with weight loss on IBD outcomes in children and young people are lacking. The European Society for Clinical Nutrition and Metabolism and United European Gastroenterology (ESPEN/UEG) guidelines do not recommend using orlistat in patients with IBD due to its mechanism of action. Orlistat reduces the absorption of fat from the diet, which leads to steatorrhea and increased fecal frequency and urgency [81]. This is especially important for children and young people living with IBD as they are at higher risk of growth delay and micronutrient deficiencies [58]. More recently, with the introduction of glucagon‐like peptide‐1 receptor agonist (GLP‐1 RA), this is offering a new treatment option for people living with obesity [82] and IBD. Evidence has shown that the use of GLP‐1 RA in adults living with Type 2 diabetes and/or obesity and IBD was associated with significant weight loss, lower risk of adverse clinical events, and improved outcomes in IBD while being shown to be safe with mild side effects [83, 84, 85, 86]. At present, data is dearth on their use within children and young people; therefore, caution should be taken at present for their use.

Studies on the effects of bariatric surgery associated with weight loss and outcomes of IBD in young people are lacking even though studies on adults generally suggest the safety [87] and favorable anti‐inflammatory effect of bariatric surgeries on IBD outcomes through the reduction in adipose tissue and levels of inflammatory markers [88]. On the other hand, there is some evidence that suggests the association of bariatric surgery with increased risk of new‐onset IBD [89] possibly through dysbiosis associated with roux‐en‐Y gastric bypass (RYGB) specifically as compared to sleeve gastrectomy (SG) [90] and reduced bile acids associated with bacterial overgrowth and inflammation [91]. It is important to mention though that the increased risk of IBD development among individuals who have undergone bariatric surgery was observed in those who were still living with obesity, whereas those who achieved a healthy BMI within 1 year after bariatric surgery were at reduced risk for IBD. This suggests that the risk of de‐novo IBD development associated with bariatric surgery in some studies might actually be due to people continuing to live with obesity rather than bariatric surgery [92]. Due to the conflicting findings, more studies are needed to confirm the association between bariatric surgery and IBD, though from the available data SG might be the preferred bariatric surgery option due to the possible consequences of having RYGB in this population [93].

## 12 Conclusion and Future Recommendations

Inflammation forms the base of IBD pathogenesis and pathophysiology, which means that factors linked to its exacerbation including overweight and obesity might also impact the clinical course of the disease. With the parallel increase seen in obesity and IBD, it has been suggested that living with overweight and obesity might influence IBD development, clinical course, and management through altered microbiota and proinflammatory effects via the secretion of hormones and inflammatory cytokines from adipose tissue. These lead to low‐grade chronic inflammation and increased intestinal permeability, which have been linked with IBD. However, clinical evidence in children and young people is still scarce and conflicting. More studies are needed to understand the underlying mechanisms and impact of overweight and obesity on the development of different IBD phenotypes among various age groups, particularly children and young people, as most studies suggest that excess weight is associated with CD development in adults, although it is more likely to be associated with UC in children and young people.

Children and young people with overweight or obesity and IBD are shown to be at risk of having decreased LM, which might increase their risk of developing SO and consequently worse outcomes of IBD. Identifying sarcopenia in children and young people living with IBD and obesity might be more challenging, as nutritional problems may not be identified by obtaining simple measures, such as weight and height, and this was especially noticed in children and young people living with UC [94]. The identification of sarcopenia requires immediate intervention due to its adverse effects on clinical outcomes in patients with IBD [60]. There is limited evidence regarding the appropriate nutritional management of SO in individuals with IBD. However, low‐calorie diets should be avoided due to their increased risk of muscle loss and nutrient deficiencies, especially during periods of relapse where the body is in a catabolic state [95]. This is even more important in cases of children and young people where IBD has adverse effects on growth [58]. Moreover, the main treatment of sarcopenia includes ensuring adequate protein intake and resistance exercise [96].

It should be noted that many of the current practice recommendations regarding children and young people with IBD, provided in a recent position paper published by the IBD Group of the European Society for Pediatric Gastroenterology, Hepatology and Nutrition (ESPGHAN), are based on studies of adults or are based on practice recommendations due to lacking evidence [97]. Furthermore, it is essential to highlight that living with overweight and obesity can alter the body's metabolism. Therefore, children and young people living with obesity might have different levels or requirements of micronutrients than those with healthy or reduced weight. Thus, more studies are needed to establish evidence‐based recommendations tailored to children and young people diagnosed with IBD and living with overweight or obesity. Routine assessment of nutritional status and body composition should be an integral part of the healthcare provided by gastroenterologists and dietitians to individuals diagnosed with IBD including children and young people living with overweight or obesity.

The effects of overweight and obesity on the clinical course and management of IBD among children and young people are still not fully clear. Weight loss interventions in children and young people including dietary and lifestyle approaches, pharmacotherapy, and bariatric surgery should be investigated due to the lacking evidence among this population; evidence remains inconclusive regarding the benefits of weight loss on IBD. Despite the conflicting literature, some studies have shown a positive impact of weight loss interventions on the outcomes of IBD in adults. Prospective studies with longer follow‐up periods and larger sample sizes are needed to clarify their effects. Due to the suggested adverse effects of increased weight on the clinical course of IBD, overweight and obesity interventions should be considered for all children and young people living with excess weight and IBD.

In conclusion, the parallel increase seen in the prevalence of overweight and obesity and IBD in children and young people and their possible association highlights the importance of investigating the underlying mechanisms of excess weight on the clinical course and outcomes of IBD. This will contribute to a better understanding of this relationship and assist in guiding effective tailored interventions, which will help improve the health and quality of life of children and young people living with overweight or obesity and IBD.

## Conflicts of Interest

A.B. declares researcher‐led grants from the National Institute for Health Research, Rosetrees Trust, MRC, INNOVATE UK, British Dietetic Association, British Association of Parenteral and Enteral Nutrition, BBRSC, the Office of Health Improvement and Disparities, and NovoNordisk. A.B. reports honoraria from Novo Nordisk and Eli Lilly outside the submitted work and is on the Medical Advisory Board and a shareholder of Reset Health Clinics Ltd. E.P. declares researcher‐led grants from the British Dietetic Association and declares no conflicts of interest. R.A. declares no conflicts of interest.

## Data Availability

Data sharing not applicable to this article as no datasets were generated or analysed during the current study.

## References

[1] D. C. D. Baumgart and S. R. P. Carding , “Inflammatory Bowel Disease: Cause and Immunobiology,” Lancet 369, no. 9573 (2007): 1627–1640, 10.1016/S0140-6736(07)60750-8. 17499605

[2] M. E. Kuenzig , S. G. Fung , L. Marderfeld , et al., “Twenty‐First Century Trends in the Global Epidemiology of Pediatric‐Onset Inflammatory Bowel Disease: Systematic Review,” Gastroenterology 162, no. 4 (2022): 1147–1159, 10.1053/j.gastro.2021.12.282. 34995526

[3] T. J. Pasvol , L. Horsfall , S. Bloom , et al., “Incidence and Prevalence of Inflammatory Bowel Disease in UK Primary Care: A Population‐Based Cohort Study,” BMJ Open 10, no. 7 (2020): e036584, 10.1136/bmjopen-2019-036584. PMC7371214 32690524

[4] J. J. Ashton , M. Cullen , N. A. Afzal , T. Coelho , A. Batra , and R. M. Beattie , “Is the Incidence of Paediatric Inflammatory Bowel Disease Still Increasing?,” Archives of Disease in Childhood 103, no. 11 (2018): 1093–1094, 10.1136/archdischild-2018-315038. 29519945

[5] T. Suzuki , Y. Sasahara , A. Kikuchi , et al., “Targeted Sequencing and Immunological Analysis Reveal the Involvement of Primary Immunodeficiency Genes in Pediatric IBD: A Japanese Multicenter Study,” Journal of Clinical Immunology 37, no. 1 (2017): 67–79, 10.1007/s10875-016-0339-5. 27747465

[6] H. H. Uhlig , “Monogenic Diseases Associated With Intestinal Inflammation: Implications for the Understanding of Inflammatory Bowel Disease,” Gut 62, no. 12 (2013): 1795–1805, 10.1136/gutjnl-2012-303956. 24203055

[7] J. J. Ashton , F. M. Barakat , C. Barnes , et al., “Incidence and Prevalence of Paediatric Inflammatory Bowel Disease Continues to Increase in the South of England,” Journal of Pediatric Gastroenterology and Nutrition 75, no. 2 (2022): e20–e24, 10.1097/MPG.0000000000003511. 35666860

[8] N. H. Phelps , R. K. Singleton , B. Zhou , et al., “Worldwide Trends in Underweight and Obesity From 1990 to 2022: A Pooled Analysis of 3663 Population‐Representative Studies With 222 Million Children, Adolescents, and Adults,” Lancet 403 (2024): 1027–1050, 10.1016/s0140-6736(23)02750-2. 38432237 PMC7615769

[9] J. A. Kerr , G. C. Patton , K. I. Cini , et al., “Global, Regional, and National Prevalence of Child and Adolescent Overweight and Obesity, 1990–2021, With Forecasts to 2050: A Forecasting Study for the Global Burden of Disease Study 2021,” Lancet 405, no. 10481 (2025): 785–812, 10.1016/S0140-6736(25)00397-6. 40049185 PMC11920006

[10] S. Singh , P. S. Dulai , A. Zarrinpar , S. Ramamoorthy , and W. J. Sandborn , “Obesity in IBD: Epidemiology, Pathogenesis, Disease Course and Treatment Outcomes,” Nature Reviews Gastroenterology & Hepatology 14, no. 2 (2017): 110–121, 10.1038/nrgastro.2016.181. 27899815 PMC5550405

[11] T. Nic Suibhne , T. C. Raftery , O. McMahon , C. Walsh , C. O'Morain , and M. O'Sullivan , “High Prevalence of Overweight and Obesity in Adults With Crohn's Disease: Associations With Disease and Lifestyle Factors,” Journal of Crohn's and Colitis 7, no. 7 (2013): e241–e248, 10.1016/j.crohns.2012.09.009. 23040290

[12] A. Jain , J. Bricker , M. D. Kappelman , and J. L. Dotson , “Overweight and Obese Status Is Not Associated With Disease Activity for Children and Adolescents With Newly Diagnosed Inflammatory Bowel Disease,” American Journal of Gastroenterology 117, no. 7 (2022): 1146–1153, 10.14309/ajg.0000000000001803. 35470288

[13] S. M. D. Kugathasan , J. B. A. Nebel , J. A. M. D. Skelton , et al., “Body Mass Index in Children With Newly Diagnosed Inflammatory Bowel Disease: Observations From Two Multicenter North American Inception Cohorts,” Journal of Pediatrics 151, no. 5 (2007): 523–527, 10.1016/j.jpeds.2007.04.004. 17961699

[14] M. D. Long , W. V. Crandall , I. H. Leibowitz , et al., “Prevalence and Epidemiology of Overweight and Obesity in Children With Inflammatory Bowel Disease,” Inflammatory Bowel Diseases 17, no. 10 (2011): 2162–2168, 10.1002/ibd.21585. 21910178 PMC3116044

[15] A. Pituch‐Zdanowska , A. Banaszkiewicz , M. Dziekiewicz , et al., “Overweight and Obesity in Children With Newly Diagnosed Inflammatory Bowel Disease,” Advances in Medical Sciences 61, no. 1 (2016): 28–31, 10.1016/j.advms.2015.07.004. 26355738

[16] M. El Mouzan , N. Alahmadi , K. A. Alsaleeem , A. Assiri , B. AlSaleem , and A. Al Sarkhy , “Prevalence of Nutritional Disorders in Saudi Children With Inflammatory Bowel Disease Based on the National Growth Reference,” Arab Journal of Gastroenterology 21, no. 3 (2020): 179–182, 10.1016/j.ajg.2020.07.002. 32798189

[17] F. R. M. Carbonell and O. C. Chandan , “Body Mass Index at Presentation of Inflammatory Bowel Disease in Children,” Pediatric Gastroenterology, Hepatology & Nutrition 23, no. 5 (2020): 439–446, 10.5223/PGHN.2020.23.5.439. PMC7481061 32953639

[18] A. Chandrakumar , A. Wang , K. Grover , and W. El‐Matary , “Obesity Is More Common in Children Newly Diagnosed With Ulcerative Colitis as Compared to Those With Crohn Disease,” Journal of Pediatric Gastroenterology and Nutrition 70, no. 5 (2020): 593–597, 10.1097/MPG.0000000000002639. 31977953

[19] A. Yerushalmy‐Feler , T. Galai , H. Moran‐Lev , et al., “BMI in the Lower and Upper Quartiles at Diagnosis and at 1‐Year Follow‐Up Is Significantly Associated With Higher Risk of Disease Exacerbation in Pediatric Inflammatory Bowel Disease,” European Journal of Pediatrics 180, no. 1 (2021): 21–29, 10.1007/s00431-020-03697-2. 32500205

[20] T. von Graffenried , A. M. Schoepfer , J.‐B. Rossel , et al., “Impact of Overweight and Obesity on Disease Outcome in the Pediatric Swiss Inflammatory Bowel Disease Cohort,” JPGN Reports 3, no. 2 (2022): e193, 10.1097/PG9.0000000000000193. 37168919 PMC10158416

[21] L. Räisänen , S. Lommi , E. Engberg , K. L. Kolho , and H. Viljakainen , “Central Obesity in School‐Aged Children Increases the Likelihood of Developing Paediatric Autoimmune Diseases,” Pediatric Obesity 17, no. 3 (2022): e12857, 10.1111/ijpo.12857. 34608761 PMC9285017

[22] H. Khalili , A. N. Ananthakrishnan , G. G. Konijeti , et al., “Measures of Obesity and Risk of Crohn's Disease and Ulcerative Colitis,” Inflammatory Bowel Diseases 21, no. 2 (2015): 361–368, 10.1097/MIB.0000000000000283. 25563694 PMC4308549

[23] A. Yerushalmy‐Feler , A. Ben‐Tov , Y. Weintraub , et al., “High and Low Body Mass Index May Predict Severe Disease Course in Children With Inflammatory Bowel Disease,” Scandinavian Journal of Gastroenterology 53, no. 6 (2018): 708–713, 10.1080/00365521.2018.1464595. 29688090

[24] D. A. Winer , H. Luck , S. Tsai , and S. Winer , “The Intestinal Immune System in Obesity and Insulin Resistance,” Cell Metabolism 23, no. 3 (2016): 413–426, 10.1016/j.cmet.2016.01.003. 26853748

[25] A. Kim , “Dysbiosis: A Review Highlighting Obesity and Inflammatory Bowel Disease,” Journal of Clinical Gastroenterology 49, no. Suppl 1 (2015): S20–S24, 10.1097/MCG.0000000000000356. 26447959

[26] G. Ecklu‐Mensah , J. Gilbert , and S. Devkota , “Dietary Selection Pressures and Their Impact on the Gut Microbiome,” Cellular and Molecular Gastroenterology and Hepatology 13, no. 1 (2022): 7–18, 10.1016/j.jcmgh.2021.07.009. 34329765 PMC8600059

[27] W. Kopp , “How Western Diet and Lifestyle Drive the Pandemic of Obesity and Civilization Diseases,” Diabetes, Metabolic Syndrome and Obesity: Targets and Therapy 12 (2019): 2221–2236, 10.2147/DMSO.S216791. 31695465 PMC6817492

[28] S. Jarmakiewicz‐czaja , A. Sokal , and R. Filip , “What Was First, Obesity or Inflammatory Bowel Disease? What Does the Gut Microbiota Have to Do With It?,” Nutrients 12, no. 10 (2020): 1–16, 10.3390/nu12103073. PMC7600052 33050109

[29] Y. Qin , J. D. Roberts , S. A. Grimm , et al., “An Obesity‐Associated Gut Microbiome Reprograms the Intestinal Epigenome and Leads to Altered Colonic Gene Expression,” Genome Biology 19, no. 1 (2018): 7, 10.1186/s13059-018-1389-1. 29361968 PMC5782396

[30] J. Laster , S. L. Bonnes , and J. Rocha , “Increased Use of Emulsifiers in Processed Foods and the Links to Obesity,” Current Gastroenterology Reports 21, no. 11 (2019): 61, 10.1007/s11894-019-0723-4. 31792622

[31] B. Chassaing , T. Van de Wiele , J. De Bodt , M. Marzorati , and A. T. Gewirtz , “Dietary Emulsifiers Directly Alter Human Microbiota Composition and Gene Expression Ex Vivo Potentiating Intestinal Inflammation,” Gut 66, no. 8 (2017): 1414–1427, 10.1136/gutjnl-2016-313099. 28325746 PMC5940336

[32] V. Svolos , H. Gordon , M. C. E. Lomer , et al., “ECCO Consensus on Dietary Management of Inflammatory Bowel Disease,” Journal of Crohn's and Colitis 19, no. 9 (2025): 1–40, 10.1093/ecco-jcc/jjaf122. 40650933

[33] X. Bian , L. Chi , B. Gao , P. Tu , H. Ru , and K. Lu , “The Artificial Sweetener Acesulfame Potassium Affects the Gut Microbiome and Body Weight Gain in CD‐1 Mice,” PLoS ONE 12, no. 6 (2017): e0178426, 10.1371/journal.pone.0178426. 28594855 PMC5464538

[34] B. Shum and S. Georgia , “The Effects of Non‐Nutritive Sweetener Consumption in the Pediatric Populations: What We Know, What We Don't, and What We Need to Learn,” Front Endocrinol 12 (2021): 625415, 10.3389/fendo.2021.625415. PMC8049500 33868167

[35] X. Bian , L. Chi , B. Gao , P. Tu , H. Ru , and K. Lu , “Gut Microbiome Response to Sucralose and Its Potential Role in Inducing Liver Inflammation in Mice,” Frontiers in Physiology 8 (2017): 487, 10.3389/fphys.2017.00487. 28790923 PMC5522834

[36] E. D. Sonnenburg , S. A. Smits , M. Tikhonov , S. K. Higginbottom , N. S. Wingreen , and J. L. Sonnenburg , “Diet‐Induced Extinctions in the Gut Microbiota Compound Over Generations,” Nature 529, no. 7585 (2016): 212–215, 10.1038/nature16504. 26762459 PMC4850918

[37] R. Sigall Boneh , C. Westoby , I. Oseran , et al., “The Crohn's Disease Exclusion Diet: A Comprehensive Review of Evidence, Implementation Strategies, Practical Guidance, and Future Directions,” Inflammatory Bowel Diseases 30, no. 10 (2024): 1888–1902, 10.1093/ibd/izad255. 37978895 PMC11446999

[38] M. J. Bull and N. T. Plummer , “Part 1: The Human Gut Microbiome in Health and Disease,” Integrative Medicine (Encinitas, California) 13, no. 6 (2014): 17–22. PMC4566439 26770121

[39] D. Rothschild , O. Weissbrod , E. Barkan , et al., “Environment Dominates Over Host Genetics in Shaping Human Gut Microbiota,” Nature (London) 555, no. 7695 (2018): 210–228, 10.1038/nature25973. 29489753

[40] O. A. Baothman , M. A. Zamzami , I. Taher , J. Abubaker , and M. Abu‐Farha , “The Role of Gut Microbiota in the Development of Obesity and Diabetes,” Lipids in Health and Disease 15, no. 1 (2016): 108, 10.1186/s12944-016-0278-4. 27317359 PMC4912704

[41] Z. Puljiz , M. Kumric , J. Vrdoljak , et al., “Obesity, Gut Microbiota, and Metabolome: From Pathophysiology to Nutritional Interventions,” Nutrients 15, no. 10 (2023): 2236, 10.3390/nu15102236. 37242119 PMC10223302

[42] M. Ferrer , A. Ruiz , F. Lanza , et al., “Microbiota From the Distal Guts of Lean and Obese Adolescents Exhibit Partial Functional Redundancy Besides Clear Differences in Community Structure: Metaproteomic Insights Associated to Human Obesity,” Environmental Microbiology 15, no. 1 (2013): 211–226, 10.1111/j.1462-2920.2012.02845.x. 22891823

[43] M. Goffredo , E. Parks , D. Wagner , K. Mass , J. Graf , and N. Santoro , “Role of Gut Microbiota and Short Chain Fatty Acids in Modulating Energy Harvest and Fat Partitioning in Youth,” Journal of Clinical Endocrinology & Metabolism 101, no. 11 (2016): 4367–4376. 27648960 10.1210/jc.2016-1797 PMC5095239

[44] W. A. Walters , Z. Xu , and R. Knight , “Meta‐Analyses of Human Gut Microbes Associated With Obesity and IBD,” FEBS Letters 588, no. 22 (2014): 4223–4233, 10.1016/j.febslet.2014.09.039. 25307765 PMC5050012

[45] J. Bai , Y. Hu , and D. W. Bruner , “Composition of Gut Microbiota and Its Association With Body Mass Index and Lifestyle Factors in a Cohort of 7–18 Years Old Children From the American Gut Project,” Pediatric Obesity 14, no. 4 (2019): e12480, 10.1111/ijpo.12480. 30417607

[46] A. Moya‐Pérez , A. Neef , Y. Sanz , and P. V. Nerurkar , “ *Bifidobacterium pseudocatenulatum* CECT 7765 Reduces Obesity‐Associated Inflammation by Restoring the Lymphocyte‐Macrophage Balance and Gut Microbiota Structure in High‐Fat Diet‐Fed Mice,” PLoS ONE 10, no. 7 (2015): e0126976, 10.1371/journal.pone.0126976. 26161548 PMC4498624

[47] K. L. Glassner , B. P. Abraham , and E. M. M. Quigley , “The Microbiome and Inflammatory Bowel Disease,” Journal of Allergy and Clinical Immunology 145, no. 1 (2020): 16–27, 10.1016/j.jaci.2019.11.003. 31910984

[48] C. Olbjørn , M. C. Småstuen , E. Thiis‐Evensen , et al., “Fecal Microbiota Profiles in Treatment‐Naïve Pediatric Inflammatory Bowel Disease – Associations With Disease Phenotype, Treatment, and Outcome,” Clinical and Experimental Gastroenterology 12 (2019): 37–49, 10.2147/CEG.S186235. 30774408 PMC6362922

[49] P. D. Cani , J. Amar , M. A. Iglesias , et al., “Metabolic Endotoxemia Initiates Obesity and Insulin Resistance,” Diabetes 56, no. 7 (2007): 1761–1772, 10.2337/db06-1491. 17456850

[50] E. Gäbele , K. Dostert , C. Hofmann , et al., “DSS Induced Colitis Increases Portal LPS Levels and Enhances Hepatic Inflammation and Fibrogenesis in Experimental NASH,” Journal of Hepatology 55, no. 6 (2011): 1391–1399, 10.1016/j.jhep.2011.02.035. 21703208

[51] J. B. Ewaschuk , H. Diaz , L. Meddings , et al., “Secreted Bioactive Factors From *Bifidobacterium infantis* Enhance Epithelial Cell Barrier Function,” American Journal of Physiology ‐ Gastrointestinal and Liver Physiology 295, no. 5 (2008): G1025–G1034, 10.1152/ajpgi.90227.2008. 18787064

[52] S. Gesta , Y.‐H. Tseng , and C. R. Kahn , “Developmental Origin of Fat: Tracking Obesity to Its Source,” Cell 131, no. 2 (2007): 242–256, 10.1016/j.cell.2007.10.004. 17956727

[53] L. I. Kredel and B. Siegmund , “Adipose‐Tissue and Intestinal Inflammation – Visceral Obesity and Creeping Fat,” Frontiers in Immunology 5 (2014): 462, 10.3389/fimmu.2014.00462. 25309544 PMC4174117

[54] J. Bilski , A. Mazur‐Bialy , D. Wojcik , et al., “Role of Obesity, Mesenteric Adipose Tissue, and Adipokines in Inflammatory Bowel Diseases,” Biomolecules (Basel, Switzerland) 9, no. 12 (2019): 780, 10.3390/biom9120780. PMC6995528 31779136

[55] J. L. Seminerio , I. E. Koutroubakis , C. Ramos‐Rivers , et al., “Impact of Obesity on the Management and Clinical Course of Patients With Inflammatory Bowel Disease,” Inflammatory Bowel Diseases 21, no. 12 (2015): 2857–2863, 10.1097/MIB.0000000000000560. 26241001

[56] J. Meddings , “What Role Does Intestinal Permeability Have in IBD Pathogenesis?,” Inflammatory Bowel Diseases 14, no. S2 (2008): S138–S139, 10.1002/ibd.20719. 18816777

[57] A. J. Cruz‐Jentoft , G. Bahat , J. Bauer , et al., “Sarcopenia: Revised European Consensus on Definition and Diagnosis,” Age and Ageing 48, no. 1 (2019): 16–31, 10.1093/ageing/afy169. 30312372 PMC6322506

[58] L. P. D. Valentini , L. M. D. Schaper , C. M. D. Buning , et al., “Malnutrition and Impaired Muscle Strength in Patients With Crohn's Disease and Ulcerative Colitis in Remission,” Nutrition 24, no. 7 (2008): 694–702, 10.1016/j.nut.2008.03.018. 18499398

[59] S. Liu , X. Ding , G. Maggiore , et al., “Sarcopenia Is Associated With Poor Clinical Outcomes in Patients With Inflammatory Bowel Disease: A Prospective Cohort Study,” Annals of Translational Medicine 10, no. 6 (2022): 367, 10.21037/atm-22-1126. 35433981 PMC9011317

[60] D. W. Adams , S. Gurwara , H. J. Silver , et al., “Sarcopenia Is Common in Overweight Patients With Inflammatory Bowel Disease and May Predict Need for Surgery,” Inflammatory Bowel Diseases 23, no. 7 (2017): 1182–1186, 10.1097/MIB.0000000000001128. 28410342

[61] C. Hartman , R. Eliakim , and R. Shamir , “Nutritional Status and Nutritional Therapy in Inflammatory Bowel Diseases,” World Journal of Gastroenterology 15, no. 21 (2009): 2570–2578, 10.3748/wjg.15.2570. 19496185 PMC2691486

[62] A. Erős , A. Soós , P. Hegyi , et al., “Sarcopenia as an Independent Predictor of the Surgical Outcomes of Patients With Inflammatory Bowel Disease: A Meta‐Analysis,” Surgery Today 50, no. 10 (2020): 1138–1150, 10.1007/s00595-019-01893-8. 31617016 PMC7501129

[63] D. Thangarajah , M. J. Hyde , V. K. S. Konteti , S. Santhakumaran , G. Frost , and J. M. E. Fell , “Systematic Review: Body Composition in Children With Inflammatory Bowel Disease,” Alimentary Pharmacology & Therapeutics 42, no. 2 (2015): 142–157, 10.1111/apt.13218. 26043941

[64] X. Jiao , W. Y. Wu , S. F. Zhan , J. B. Liu , and X. J. Zhang , “A Bidirectional Mendelian Randomization Study of Sarcopenia‐Related Traits and Inflammatory Bowel Diseases,” Frontiers in Immunology 14 (2023): 1240811, 10.3389/fimmu.2023.1240811. 38022582 PMC10666781

[65] G. Huppertz‐Hauss , M. L. Høivik , E. Langholz , et al., “Health‐Related Quality of Life in Inflammatory Bowel Disease in a European‐Wide Population‐Based Cohort 10 Years After Diagnosis,” Inflammatory Bowel Diseases 21, no. 2 (2015): 337–344, 10.1097/mib.0000000000000272. 25569735 PMC4345967

[66] J. J. McGing , S. J. Radford , S. T. Francis , S. Serres , P. L. Greenhaff , and G. W. Moran , “Review Article: The Aetiology of Fatigue in Inflammatory Bowel Disease and Potential Therapeutic Management Strategies,” Alimentary Pharmacology & Therapeutics 54, no. 4 (2021): 368–387, 10.1111/apt.16465. 34228817

[67] C. B. Turer , H. Lin , and G. Flores , “Prevalence of Vitamin D Deficiency Among Overweight and Obese US Children,” Pediatrics 131, no. 1 (2013): e152–e161, 10.1542/peds.2012-1711. 23266927

[68] D. Gilbert‐Diamond , A. Baylin , M. Mora‐Plazas , et al., “Vitamin D Deficiency and Anthropometric Indicators of Adiposity in School‐Age Children: A Prospective Study,” American Journal of Clinical Nutrition 92, no. 6 (2010): 1446–1451, 10.3945/ajcn.2010.29746. 20926524 PMC3131841

[69] R. J. Wood , “Vitamin D and Adipogenesis: New Molecular Insights,” Nutrition Reviews 66, no. 1 (2008): 40–46, 10.1111/j.1753-4887.2007.00004.x. 18254883

[70] S. Ehrlich , A. G. Mark , F. Rinawi , R. Shamir , and A. Assa , “Micronutrient Deficiencies in Children With Inflammatory Bowel Diseases,” Nutrition in Clinical Practice 35, no. 2 (2020): 315–322, 10.1002/ncp.10373. 31342601

[71] H. V. Lin , A. Frassetto , E. J. Kowalik, Jr. , et al., “Butyrate and Propionate Protect Against Diet‐Induced Obesity and Regulate Gut Hormones via Free Fatty Acid Receptor 3‐Independent Mechanisms,” PLoS ONE 7, no. 4 (2012): e35240, 10.1371/journal.pone.0035240. 22506074 PMC3323649

[72] O. Kadenczki , A. Dezsofi , A. Cseh , et al., “Disease Activity Is Associated With Obesity in Newly Diagnosed Pediatric Patients With Ulcerative Colitis,” International Journal of Environmental Research and Public Health 19, no. 23 (2022): 16091, 10.3390/ijerph192316091. 36498163 PMC9738058

[73] N. P. Zwintscher , J. D. Horton , and S. R. Steele , “Obesity Has Minimal Impact on Clinical Outcomes in Children With Inflammatory Bowel Disease,” Journal of Pediatric Surgery 49, no. 2 (2014): 265–268, 10.1016/j.jpedsurg.2013.11.033. 24528963

[74] S. Sila , M. Aloi , U. Cucinotta , et al., “Effect of Overweight and Obesity on the Response to Anti‐TNF Therapy and Disease Course in Children With IBD,” Inflammatory Bowel Diseases 31 (2024): 1263–1271, 10.1093/ibd/izae165. 39083286

[75] D. R. Ebach , T. W. Jester , J. A. Galanko , et al., “High Body Mass Index and Response to Anti‐Tumor Necrosis Factor Therapy in Pediatric Crohn's Disease,” American Journal of Gastroenterology 119, no. 6 (2024): 1110–1116, 10.14309/ajg.0000000000002741. 38445644 PMC11150092

[76] C. Lejus , G. Orliaguet , F. Servin , et al., “Peri‐Operative Management of Overweight and Obese Children and Adolescents,” Lancet Child & Adolescent Health 1, no. 4 (2017): 311–322, 10.1016/S2352-4642(17)30090-1. 30169186

[77] L. Haas , R. Chevalier , B. T. Major , F. Enders , S. Kumar , and J. Tung , “Biologic Agents Are Associated With Excessive Weight Gain in Children With Inflammatory Bowel Disease,” Digestive Diseases and Sciences 62, no. 11 (2017): 3110–3116, 10.1007/s10620-017-4745-1. 28895012

[78] J. H. Kim and J. H. Yoo , “Obesity and Novel Management of Inflammatory Bowel Disease,” World Journal of Gastroenterology 29, no. 12 (2023): 1779–1794, 10.3748/wjg.v29.i12.1779. 37032724 PMC10080699

[79] S. C. Bischoff , P. Bager , J. Escher , et al., “ESPEN Guideline on Clinical Nutrition in Inflammatory Bowel Disease,” Clinical Nutrition (Edinburgh, Scotland) 42, no. 3 (2023): 352–379, 10.1016/j.clnu.2022.12.004. 36739756

[80] O. T. Mytton , D. Nicholls , S. Saxena , and R. M. Viner , “Approach to a Child or Young Person With Concerns About Excess Weight,” BMJ 380 (2023): e074255, 10.1136/bmj-2022-074255. 36796840

[81] NHS , “Treatment‐Obesity,” https://www.nhs.uk/conditions/obesity/treatment/.

[82] A. Brown , D. Mellor , J. Makaronidis , E. Shuttlewood , A. D. Miras , and D. J. Pournaras , ““From Evidence to Practice” – Insights From the Multidisciplinary Team on the Optimal Integration of GLP‐1 Receptor Agonists in Obesity Management Services,” Nutrition Bulletin 49, no. 3 (2024): 257–263, 10.1111/nbu.12700. 39136338

[83] I. Levine , S. Sekhri , W. Schreiber‐Stainthorp , et al., “GLP‐1 Receptor Agonists Confer No Increased Rates of IBD Exacerbation Among Patients With IBD,” Inflammatory Bowel Diseases 31 (2024): 467–475, 10.1093/ibd/izae250. PMC12476976 39438251

[84] M. Villumsen , A. B. Schelde , E. Jimenez‐Solem , T. Jess , and K. H. Allin , “GLP‐1 Based Therapies and Disease Course of Inflammatory Bowel Disease,” EClinicalMedicine 37 (2021): 100979, 10.1016/j.eclinm.2021.100979. 34386751 PMC8343256

[85] Y. Gorelik , I. Ghersin , R. Lujan , et al., “GLP‐1 Analog Use Is Associated With Improved Disease Course in Inflammatory Bowel Disease: A Report From the Epi‐IIRN,” Journal of Crohn's and Colitis 19, no. 4 (2024): 1–6, 10.1093/ecco-jcc/jjae160. 39441993

[86] C. Ramos Belinchón , H. Martínez‐Lozano , C. Serrano Moreno , et al., “Effectiveness and Safety of GLP‐1 Agonist in Obese Patients With Inflammatory Bowel Disease,” Revista Española de Enfermedades Digestivas 116, no. 9 (2024): 478–483, 10.17235/reed.2024.10305/2024. 38767015

[87] N. P. McKenna , E. B. Habermann , A. Sada , T. A. Kellogg , and T. J. McKenzie , “Is Bariatric Surgery Safe and Effective in Patients With Inflammatory Bowel Disease?,” Obesity Surgery 30, no. 3 (2020): 882–888, 10.1007/s11695-019-04267-8. 31758472

[88] P. Sharma , T. R. McCarty , and B. Njei , “Impact of Bariatric Surgery on Outcomes of Patients With Inflammatory Bowel Disease: A Nationwide Inpatient Sample Analysis, 2004–2014,” Obesity Surgery 28, no. 4 (2018): 1015–1024, 10.1007/s11695-017-2959-0. 29047047

[89] J. Wise , T. Plescia , B. P. Cummings , and V. Lyo , “Exploring the Relationship Between Bariatric Surgery and Inflammatory Bowel Disease: A Systematic Review,” Crohn's & Colitis 360 4, no. 2 (2022): otac013, 10.1093/crocol/otac013. PMC9802289 36777046

[90] M. Kermansaravi , R. Valizadeh , B. Farazmand , et al., “De Novo Inflammatory Bowel Disease Following Bariatric Surgery: A Systematic Review and Meta‐Analysis,” Obesity Surgery 32, no. 10 (2022): 3426–3434, 10.1007/s11695-022-06226-2. 35906528

[91] N. C. Penney , J. Kinross , R. C. Newton , and S. Purkayastha , “The Role of Bile Acids in Reducing the Metabolic Complications of Obesity After Bariatric Surgery: A Systematic Review,” International Journal of Obesity 39, no. 11 (2015): 1565–1574, 10.1038/ijo.2015.115. 26081915

[92] G. S. Kochhar , A. Desai , A. Syed , et al., “Risk of De‐Novo Inflammatory Bowel Disease Among Obese Patients Treated With Bariatric Surgery or Weight Loss Medications,” Alimentary Pharmacology & Therapeutics 51, no. 11 (2020): 1067–1075, 10.1111/apt.15721. 32319111

[93] R. Garg , B. P. Mohan , S. Ponnada , et al., “Safety and Efficacy of Bariatric Surgery in Inflammatory Bowel Disease Patients: A Systematic Review and Meta‐Analysis,” Obesity Surgery 30, no. 10 (2020): 3872–3883, 10.1007/s11695-020-04729-4. 32578179

[94] R. J. Hill and P. S. W. Davies , “You Look All Right to Me: Compromised Nutritional Status in Paediatric Patients With Ulcerative Colitis,” Journal of Pediatric Gastroenterology and Nutrition 56, no. 4 (2013): 385–389, 10.1097/MPG.0b013e31827e1f25. 23201705

[95] S. C. Bischoff , R. Barazzoni , L. Busetto , et al., “European Guideline on Obesity Care in Patients With Gastrointestinal and Liver Diseases – Joint European Society for Clinical Nutrition and Metabolism/United European Gastroenterology Guideline,” United European Gastroenterology Journal 10, no. 7 (2022): 663–720, 10.1002/ueg2.12280. 35959597 PMC9486502

[96] A. J. Cruz‐Jentoft , F. Landi , S. M. Schneider , et al., “Prevalence of and Interventions for Sarcopenia in Ageing Adults: A Systematic Review. Report of the International Sarcopenia Initiative (EWGSOP and IWGS),” Age and Ageing 43, no. 6 (2014): 48–759, 10.1093/ageing/afu115. PMC4204661 25241753

[97] E. Miele , R. Shamir , M. Aloi , et al., “Nutrition in Pediatric Inflammatory Bowel Disease: A Position Paper on Behalf of the Porto Inflammatory Bowel Disease Group of the European Society of Pediatric Gastroenterology, Hepatology and Nutrition,” Journal of Pediatric Gastroenterology and Nutrition 66, no. 4 (2018): 687–708, 10.1097/MPG.0000000000001896. 29570147

